# Case Series: A Minimally Invasive Tunneling Approach for Cubital Tunnel Syndrome

**DOI:** 10.7759/cureus.4540

**Published:** 2019-04-25

**Authors:** Gazi Rashid, Muhibullah S Tora, Long Di, Pavlos Texakalidis, Jessica N Bentley, Nicholas M Boulis

**Affiliations:** 1 Surgery, University of Massachusetts Medical School, Worcester, USA; 2 Neurosurgery, Emory University School of Medicine, Atlanta, USA; 3 Neurosurgery, Emory University Hospital, Atlanta, USA; 4 Neurosurgery, University of Alabama at Birmingham, Birmingham, USA

**Keywords:** cubital tunnel syndrome, cuts, ulnar neuropathy, peripheral nerve surgery, minimally invasive

## Abstract

Background

Cubital tunnel syndrome (CuTS) is the second most common peripheral neuropathy in the United States. All three current surgical treatment approaches, consisting of in situ decompression, medial epicondylectomy, and transposition, require large curvilinear incisions and dissections that cross the medial epicondyle. However, the use of a large curvilinear incision may not be necessary for in situ decompression and may be achieved with small incisions proximal and distal to the medial epicondyle. This may limit the risk of peri-incisional pain and numbness, similar to the benefits provided by endoscopy.

Objective

The aim of this study is to evaluate a minimally invasive tunneling approach for in situ ulnar nerve decompression utilizing 2 cm incisions proximal and distal to the medial epicondyle.

Methods

A retrospective chart review was performed for patients at Emory University Hospital with CuTS who underwent minimally invasive tunneling for in situ decompression. Seven cases were identified. Patient demographics and data on post-operative complications were collected. Pre-operative severity was graded as a Modified McGowan severity. The primary outcome was evaluated using the post-surgical Messina Criterion. Secondary outcomes were reports of peri-incisional pain or numbness evaluated at follow-up. Descriptive statistics are presented.

Results

Pre-operatively, one of the seven cases was Grade I McGowan and the remaining six were Grade 2a or 2b. Post-operatively, on the Messina Criterion, four of seven patients were rated as having “Good” outcomes, two of seven had “Fair”, while one of seven had “Poor.” There was one post-operative surgical site infection. Among the other six cases, there were no reports of peri-incisional pain or numbness.

Conclusions

The use of less-invasive tunneling approach to in situ decompression yielded positive outcomes in this case series with no reports of peri-incisional pain or numbness. A prospective trial may be useful to explore the theoretical benefits of this novel tunneling approach.

## Introduction

Entrapment of the ulnar nerve at the elbow, or Cubital tunnel syndrome (CuTS), affects 1% of the United States population. It is the second most common peripheral neuropathy after carpal tunnel syndrome [1]. CuTS typically has a slow and intermittent progression, starting with paresthesia in the ulnar distribution and progressing to intrinsic hand muscle weakness, clumsiness, and atrophy [2]. Diagnosis of CuTS is made by the clinical symptomatology and electrophysiology studies. Mild cases may improve with conservative measures, but total resolution is rare and the disease is usually progressive [2]. Thus, much of the literature recommends surgical treatment early in the disease course.

There are three standard surgical approaches for the treatment of CuTS: in situ decompression, medial epicondylectomy, or anterior transposition of the nerve. However, no gold standard exists among these three techniques. The literature currently reports comparable outcomes and varying rates of complications among these approaches [1]. All of these approaches require a large curvilinear incision that crosses the medial epicondyle. In addition, these open approaches can cause peri-incisional pain or numbness due to the large curvilinear incision causing damage to the median antebrachial cutaneous nerves [3]. This retrospective case series was performed to determine if a minimally invasive approach to decompression through two small incisions can achieve similar outcomes and minimize the risk of peri-incisional pain or numbness.

## Materials and methods

A retrospective chart review was performed to identify patients with CuTS between 2015 and 2018, who received minimally invasive tunneling for in situ decompression by a single surgeon. Seven cases were identified in six patients, one of whom had the procedure on both arms. The modified McGowan system (Table 1) was used to classify the severity of a patient’s ulnar neuropathy [4].

**Table 1:** Modified-McGowan Stages of Cubital Tunnel Syndrome

Grade	Description
1	Purely subjective symptoms causing dysfunction in daily activities
2A	Muscle weakness with or without subjective symptoms, without detectable atrophy
2B	Muscle weakness with or without subjective symptoms, with detectable atrophy
3	Disabling weakness, marked intrinsic atrophy, and profound sensory disturbances

The first follow-up visit was held after at least 14 days post-operatively. The primary post-operative outcome was assessed by Messina’s post-operative criteria (Table 2) [5].

**Table 2:** Messina's Post-Operative Criteria

Grade	Description
Excellent	Complete resolution of symptoms with no post-operative sensory, motor deficit
Good	General resolution of symptoms with mild residual site tenderness, decreased sensation, or motor weakness
Fair	Improvement post-operatively but persistent sensory changes, motor loss, muscle wasting, or claw deformity
Poor	No improvement after the surgical procedure or worsening symptoms

Secondary outcome measures included the presence of complications of surgical site infection, dehiscence, or any peri-incisional numbness or new signs of iatrogenic nerve injury. Descriptive statistics including percentages and standard deviation (SD) were used.

Surgical technique

**Figure 1:**
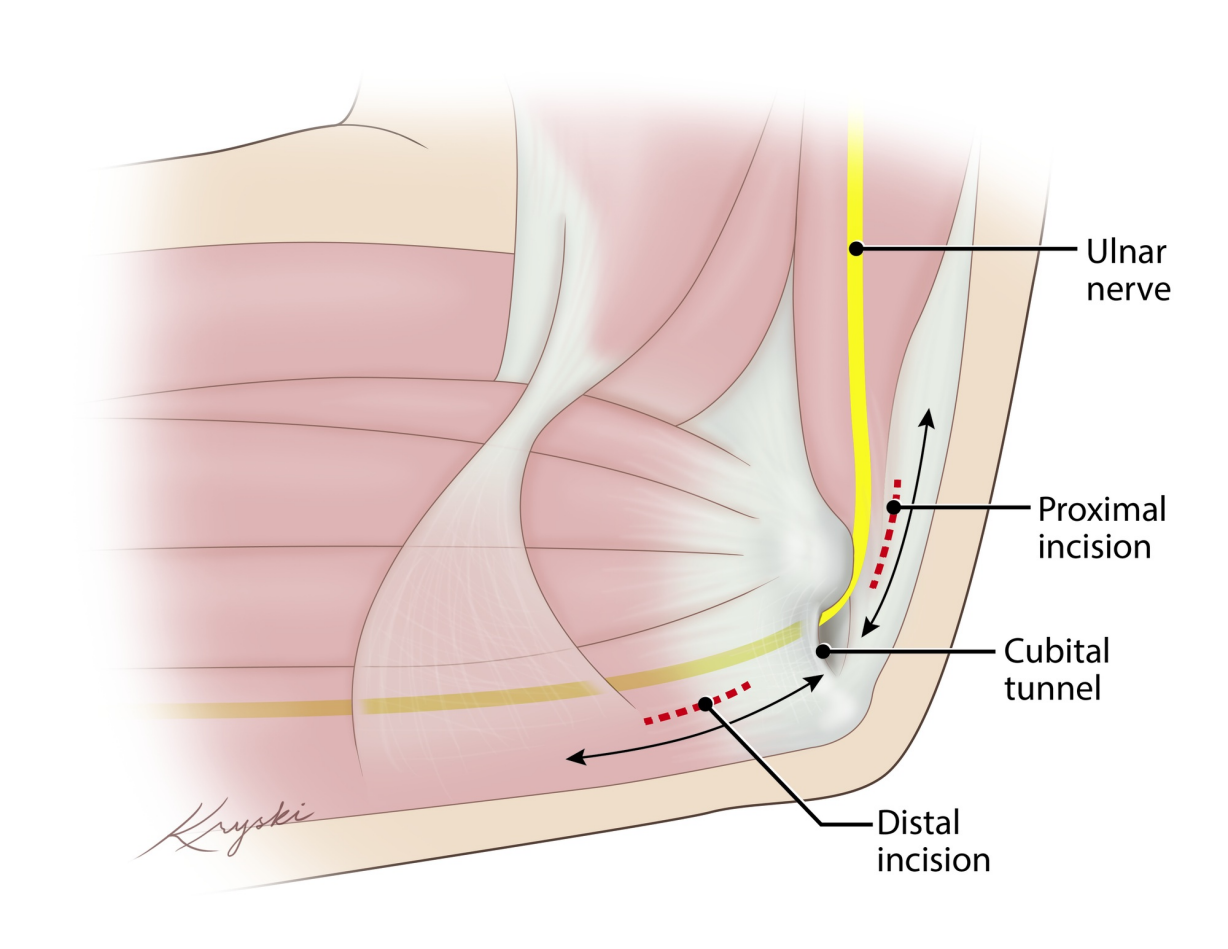
Schematic Representation of Surgical Approach Incisions proximal and distal to the cubital tunnel are made (Red-Dashes). Decompression proceeds proximally and distally from each incision site (Arrows).

Patients are brought to the operative suite, intubated, induced, and draped in a standard fashion. The affected arm is positioned on two side-by-side arm boards situated perpendicular to the bed. The arm is circumferentially prepped and draped in a standard fashion. Two 2-cm linear demarcations are made just medial to the medial epicondyle, overlying the ulnar nerve proximal and distal to the Cubital Tunnel without crossing it. At both sites, 1% lidocaine with epinephrine is administered locally. Incision sites and direction of decompression illustrated in Figure 1.

The proximal incision is made using a 10-blade and followed by electrocautery. A right-angle dissector and a mosquito are used for blunt and sharp dissection through the subcutaneous tissues to expose the ulnar nerve proximal to the Cubital Tunnel. Metzenbaum scissors are used to incise the aponeurosis and the compressive fascia overlying the ulnar nerve in the proximal tunnel with the arm fully extended. A Senn retractor is used to visualize the ulnar nerve in a fashion similar to visualizing the median during a carpal tunnel release. The Senn retractor is then used to visualize the nerve proximal to the incision, with the arm in a flexed position to keep the nerve taut. The Metzenbaum scissors are used to perform neurolysis proximally for approximately 4 cm. When neurolysis is completed the index finger can be easily inserted along the nerve. A Penfield-1 is inserted through the Cubital tunnel superficial to the nerve, protecting it.

The distal incision is made over the ulnar nerve distal to the Cubital tunnel, using a scalpel to cut down on the Penfield instrument. A self-retaining retractor is then placed in the incision and the Penfield is removed to allow visualization of the underlying nerve. The Senn retractor can then be used to view the nerve proximally, and the nerve can be safely dissected in distal Cubital tunnel with the arm in the extended position. Upon completion of this step, the nerve can be viewed free in the Cubital Tunnel. Finally, the Senn is used to elevate the superficial tissue over the distal nerve. The fascia of the flexor muscle is divided for several centimeters distal to the nerve. Both sites are probed and palpated by hand proximally and distally to locate and incise any additional septa or sites of impedance. Care is taken to preserve small branches of the ulnar nerve that were visible during the dissection. The wound is irrigated and meticulous hemostasis is obtained with the bipolar taking care not to damage or devascularize the nerve. Finally, dermis and skin are closed.

## Results

From September 2015 to February 2018, there were seven cases of CuTS in six patients. The patient group was composed of four males and two females, with an average age of 57.8 years. On the Modified McGowan Score, five of seven presented with Grade 2B severity, one with Grade 2A, and one with Grade 1.

The post-operative follow-up was performed at an average of 24.6 (SD: +/- 10.9) days post-operatively. One case had a surgical site infection and was treated with two courses of 10 days of cephalexin. None of the other six cases resulted in peri-incisional numbness, pain, new or different paresthesias, hematomas, or dehiscence. On the Messina post-operative criteria, four of seven scored “Good”, two of seven scored “Fair” and one of seven had “Poor” outcome (Table 3).

**Table 3:** Patient Characteristics and Outcomes R: Right, L: Left, CuTS: Cubital Tunnel Syndrome, UND: Ulnar Nerve Decompression.

Patient Demographics				Preoperative Characteristics			Post-Operative Characteristics			
Patient	Age at Surgery	Sex	Race	Indication	McGowan Score	Surgery	Follow-up (Days)	Messina Criterion	Complications	Peri-Incisional Pain or Numbness
1	57	Male	Caucasian	R. CuTS	2B	R. UND	37	Good	None	No
	57			L. CuTS	2A	L. UND	39	Fair	None	No
2	51	Female	Caucasian	L. CuTS	1	L. UND	31	Good	None	No
3	69	Male	Caucasian	R. CuTS	2B	R. UND	14	Poor	Peri-incisional cellulitis	Yes, Pain
4	58	Female	Caucasian	L. CuTS	2B	L. UND	16	Fair	None	No
5	50	Male	Hispanic	R. CuTS	2B	R. UND	21	Good	None	No
6	62	Male	Caucasian	L. CuTS	2B	L. UND	14	Good	None	No

## Discussion

The findings of this case series indicate a potentially promising alternative to the three standard surgical techniques for CuTS. By scoring “Good” or “Fair” on the Messina criteria, six of the seven cases were shown to have general or some resolution of symptoms after the operation. The only case to have scored “Poor” was also the only patient to have suffered a complication, a surgical site infection (SSI). However, that patient was the only one of the six known to have Type II diabetes mellitus, an established risk factor for SSIs [6]. The other six cases had no noted complications.

Among the existing surgical techniques, there is still no established gold standard. Two studies of the three standard surgical approaches to CuTS - anterior subcutaneous transposition, in situ decompression, medial epicondylectomy - have found similar rates of favorable outcomes with all three [1, 7]. According to a case series and systematic review, up to 78% of patients had post-operative improvement and the overall complication rates of all three have been shown to be 6.9%, 7.6%, and 14.5%, respectively [1]. Among baseline characteristics, young age and a better pre-operative neurologic status of McGowan Grade I were predictors of improved post-operative outcomes on uni-variate and multi-variate analysis [1]. The most common complications found after all three including peri-incisional numbness, wound dehiscence, hematomas, or signs of a new iatrogenic nerve injury. These complications, especially the paresthesias, could be related to the large curvilinear incisions required by all three.

A newer endoscopic approach has also been shown to offer slightly better outcomes and slightly lower rates of overall complications [3, 8]. However, a meta-analysis comparing open vs. endoscopic approach found that the endoscopic group had higher rates of post-operative hematomas and that both groups had similar reoperation rates [9]. Furthermore, multiple studies cite the higher cost and lower reimbursements of endoscopic approach [10] and the large variability in approaches and equipment as drawbacks [3, 11]. We believe that, like endoscopic decompression, the technique described here achieves excellent neurolysis without disruption of the superficial sensory nerves, hence reducing the risk of postoperative incisional pain, numbness, and paresthesia. Similarly, these smaller incisions heal faster than a larger curvilinear incision. While further study is required, the implementation of this less-invasive approach may be an alternative to endoscopic surgery in resource-poor settings, given the technological, training, and financial demands of the endoscopic approach.

This minimally invasive tunneling approach to in situ ulnar nerve decompression, with the high rate of “Good” or “Fair” improvement and low rate of complications, offers a promising alternative to open in situ decompression. The findings in this series warrant a larger, comparative, prospective study to determine if this approach could offer a better treatment option for patients with CuTS.

Limitations

This study did have limitations that are inherent in any retrospective case series. The McGowan Score and Messina Criteria in this study were based on clinical notes. After the first follow-up visit, long-term follow-up was heterogeneous. Finally, the study had a smaller sample size of seven cases and lacked comparison to patients who underwent the standard decompression technique.

## Conclusions

The use of less-invasive tunneling approach to in situ decompression yielded positive outcomes in this case series with no reports of peri-incisional pain or numbness. A prospective trial may be useful to explore the theoretical benefits of this novel tunneling approach.
